# Bis{μ-1-[(2-oxidophen­yl)imino­meth­yl]-2-naphtholato}bis­[pyridine­copper(II)]

**DOI:** 10.1107/S1600536809053665

**Published:** 2009-12-19

**Authors:** Kai Liu, Guihua Liu, Zhiqiang Cao, Meiju Niu

**Affiliations:** a College of Chemistry and Chemical Engineering, Liaocheng University, Shandong 252059, People’s Republic of China

## Abstract

The dinuclear title complex, [Cu_2_(C_17_H_11_NO_2_)_2_(C_5_H_5_N)_2_], consists of centrosymmetric dimers in which the Cu^II^ atom displays an elongated square-pyramidal coordination geometry. The conformation of the dimer is stabilized by inter­molecular C—H⋯O hydrogen bonds and by π–π aromatic stacking inter­actions involving the pyridine and benzene rings with centroid–centroid separations of 3.624 (3) Å.

## Related literature

For the properties and applications of Schiff bases, see: Garnovskii *et al.* (1993 ▶). For related structures, see: Zhang *et al.* (2003 ▶); Elmali *et al.* (1993 ▶).
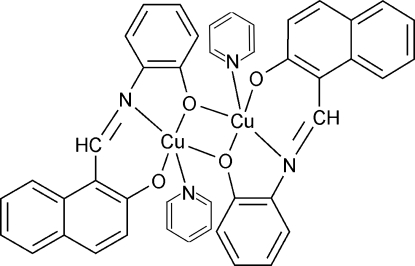


## Experimental

### 

#### Crystal data

[Cu_2_(C_17_H_11_NO_2_)_2_(C_5_H_5_N)_2_]
*M* *_r_* = 807.82
Monoclinic, 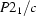

*a* = 9.4389 (8) Å
*b* = 15.8573 (17) Å
*c* = 12.0895 (15) Å
β = 105.7590 (10)°
*V* = 1741.5 (3) Å^3^
*Z* = 2
Mo *K*α radiation
μ = 1.27 mm^−1^
*T* = 298 K
0.50 × 0.26 × 0.16 mm

#### Data collection

Siemens SMART CCD area-detector diffractometer
Absorption correction: multi-scan (*SADABS*; Sheldrick, 1996 ▶) *T*_min_ = 0.568, *T*_max_ = 0.822
8621 measured reflections
3066 independent reflections
2184 reflections with *I* > 2σ(*I*)
*R*_int_ = 0.040

#### Refinement

*R*[*F* ^2^ > 2σ(*F* ^2^)] = 0.038
*wR*(*F* ^2^) = 0.092
*S* = 1.01
3066 reflections
244 parameters
H-atom parameters constrained
Δρ_max_ = 0.45 e Å^−3^
Δρ_min_ = −0.32 e Å^−3^

### 

Data collection: *SMART* (Siemens, 1996 ▶); cell refinement: *SAINT* (Siemens, 1996 ▶); data reduction: *SAINT*; program(s) used to solve structure: *SHELXS97* (Sheldrick, 2008 ▶); program(s) used to refine structure: *SHELXL97* (Sheldrick, 2008 ▶); molecular graphics: *SHELXTL* (Sheldrick, 2008 ▶); software used to prepare material for publication: *SHELXTL*.

## Supplementary Material

Crystal structure: contains datablocks I, global. DOI: 10.1107/S1600536809053665/rz2402sup1.cif

Structure factors: contains datablocks I. DOI: 10.1107/S1600536809053665/rz2402Isup2.hkl

Additional supplementary materials:  crystallographic information; 3D view; checkCIF report

## Figures and Tables

**Table 1:** Hydrogen-bond geometry (Å, °)

*D*—H⋯*A*	*D*—H	H⋯*A*	*D*⋯*A*	*D*—H⋯*A*
C18—H18⋯O1	0.93	2.31	2.872 (4)	119
C22—H22⋯O2	0.93	2.31	2.885 (4)	120

**Fig. 1.**
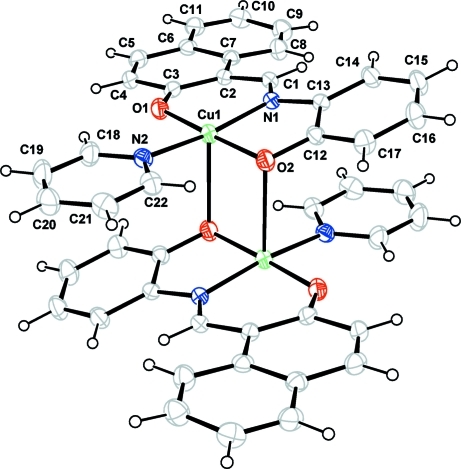
The molecular structure of the title complex with displacement ellipsoids drawn at the 30% probability level. Unlabelled atoms are related to labelled atoms by the symmetry operator (-x, 2-y, -z).

**Fig. 2.**
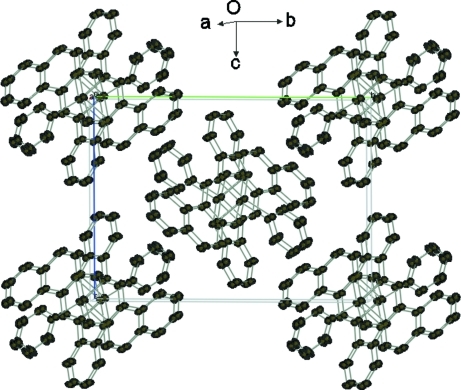
Crystal packing of the title compound viewed approximately along the a axis. Hydrogen atoms are omitted for clarity.

## supplementary crystallographic information

### Comment

Schiff bases have played an important role in coordination chemistry of transition metals, mainly due to their stability, ease of preparation, structural variability and variety of applications (Garnovskii *et al.*, 1993). Copper(II) complexes with tetradentate or tridentate N-alkylidene or N-arylidene-alkanato Schiff-base ligands are of considerable interest due to their structural and magnetic properties, in addition to being potential models for a number of important biological systems. As a continuation of our studied in this domain, we have synthesized the title complex and present its crystal structure here.

The molecular structure of the title compound is shown in Fig. 1. The compound consists of centrosymmetrical dimers where two [CuLPy] [L = 1-[(2-oxidophenyl)iminomethyl]-naphthalen-2-olato] units are linked by two bridging O2 atoms. Each copper(II) metal exhibits an elongated square pyramidal coordination geometry, with the basal plane provided by the N, O donor atoms of the tridentate L ligand and the N atom of a the pyridine molecule, and the apical position occupied by the centrosymmetrically related phenolic O2 atom (Cu1—O2^i^ = 2.5854 (19) Å; symmetry code: (i) -x, 2-y, -z). The sum of the bond angles within the basal plane is 360.16 (10)°. The Cu—O distances of 1.900 (2)Å and 1.935 (2)Å are very close to the corresponding values found in a related structure (Zhang *et al.*, 2003). The two copper(II) centres are 3.256 (2) Å apart and the distance between the two bridging O2 atoms is 3.208 (4) Å. The Cu—O(2)—Cu^i^ angle in the four-membered Cu_2_O_2_ ring is 90.99 (3)° (Ayhan & Yalon, 1993). The conformation of the dimer is stabilized by interligand C—H···O hydrogen bonds (Table 1) and by π–π aromatic stacking interactions occurring between centrosimmetrically related pyridine and benzene rings, with centroid-to-centroid separations of 3.624 (3) Å. The crystal packing (Fig. 2) is enforced only by van der Waals interactions.

### Experimental

The Schiff base C_17_H_13_NO_2_ was synthesized by condensing equimolar quantities of 2-hydroxynaphthalenaldehyde and 2-aminophenol in ethanol. Copper dichloride dihydrate (2 mmol, 341.0 mg) and the Schiff base (1 mmol, 263.3 mg) were dissolved in pyridine (22 ml). The reaction was carried out under nitrogen atmosphere. The dark brown solution was stirred for four hour and then filtered. Evaporation of the solvent yielded dark green crystals of the title compound suitable for X-ray analysis. Analysis found: C 65.47, H 4.00, N 6.92%; calculated for C_44_H_32_N4_O4_Cu_2_: C 65.42, H 3.99, N 6.94%.

### Refinement

All H atoms were placed geometrically and treated as riding on their parent atoms, with C—H = 0.93 Å and *U*_iso_(H) = 1.2 *U*_eq_(C).

### Crystal data

**Table:**

[Cu_2_(C_17_H_11_NO_2_)_2_(C_5_H_5_N)_2_]	*F*(000) = 828
*M**_r_* = 807.82	*D*_x_ = 1.541 Mg m^−^^3^
Monoclinic, *P*2_1_/*c*	Mo *K*α radiation, λ = 0.71073 Å
Hall symbol: -P 2ybc	Cell parameters from 2404 reflections
*a* = 9.4389 (8) Å	θ = 2.6–24.4°
*b* = 15.8573 (17) Å	µ = 1.27 mm^−^^1^
*c* = 12.0895 (15) Å	*T* = 298 K
β = 105.759 (1)°	Block, dark green
*V* = 1741.5 (3) Å^3^	0.50 × 0.26 × 0.16 mm
*Z* = 2	

### Data collection

**Table:**

Siemens SMART CCD area-detector diffractometer	3066 independent reflections
Radiation source: fine-focus sealed tube	2184 reflections with *I* > 2σ(*I*)
graphite	*R*_int_ = 0.040
phi and ω scans	θ_max_ = 25.0°, θ_min_ = 2.2°
Absorption correction: multi-scan (*SADABS*; Sheldrick, 1996)	*h* = −11→11
*T*_min_ = 0.568, *T*_max_ = 0.822	*k* = −18→18
8621 measured reflections	*l* = −12→14

### Refinement

**Table:**

Refinement on *F*^2^	Primary atom site location: structure-invariant direct methods
Least-squares matrix: full	Secondary atom site location: difference Fourier map
*R*[*F*^2^ > 2σ(*F*^2^)] = 0.038	Hydrogen site location: inferred from neighbouring sites
*wR*(*F*^2^) = 0.092	H-atom parameters constrained
*S* = 1.01	*w* = 1/[σ^2^(*F*_o_^2^) + (0.030*P*)^2^ + 1.6033*P*] where *P* = (*F*_o_^2^ + 2*F*_c_^2^)/3
3066 reflections	(Δ/σ)_max_ = 0.001
244 parameters	Δρ_max_ = 0.45 e Å^−^^3^
0 restraints	Δρ_min_ = −0.32 e Å^−^^3^

### Special details

**Table:**

Geometry. All e.s.d.'s (except the e.s.d. in the dihedral angle between two l.s. planes) are estimated using the full covariance matrix. The cell e.s.d.'s are taken into account individually in the estimation of e.s.d.'s in distances, angles and torsion angles; correlations between e.s.d.'s in cell parameters are only used when they are defined by crystal symmetry. An approximate (isotropic) treatment of cell e.s.d.'s is used for estimating e.s.d.'s involving l.s. planes.
Refinement. Refinement of *F*^2^ against ALL reflections. The weighted *R*-factor *wR* and goodness of fit *S* are based on *F*^2^, conventional *R*-factors *R* are based on *F*, with *F* set to zero for negative *F*^2^. The threshold expression of *F*^2^ > σ(*F*^2^) is used only for calculating *R*-factors(gt) *etc*. and is not relevant to the choice of reflections for refinement. *R*-factors based on *F*^2^ are statistically about twice as large as those based on *F*, and *R*- factors based on ALL data will be even larger.

### Fractional atomic coordinates and isotropic or equivalent isotropic displacement parameters (Å^2^)

**Table:**

	*x*	*y*	*z*	*U*_iso_*/*U*_eq_	
O2	0.0610 (2)	1.05392 (13)	0.11510 (18)	0.0401 (6)	
Cu1	0.15664 (4)	1.03970 (2)	−0.00642 (3)	0.03617 (15)	
N1	0.2782 (3)	0.95821 (16)	0.0972 (2)	0.0312 (6)	
N2	0.0524 (3)	1.14158 (16)	−0.0918 (2)	0.0374 (7)	
O1	0.2546 (3)	1.01949 (14)	−0.12192 (18)	0.0422 (6)	
C1	0.3687 (4)	0.9057 (2)	0.0708 (3)	0.0346 (8)	
H1	0.4185	0.8691	0.1284	0.041*	
C2	0.3996 (3)	0.8984 (2)	−0.0381 (3)	0.0335 (8)	
C3	0.3440 (4)	0.9580 (2)	−0.1268 (3)	0.0355 (8)	
C4	0.3917 (4)	0.9521 (2)	−0.2291 (3)	0.0406 (8)	
H4	0.3541	0.9899	−0.2886	0.049*	
C5	0.4894 (4)	0.8936 (2)	−0.2422 (3)	0.0438 (9)	
H5	0.5198	0.8934	−0.3092	0.053*	
C6	0.5471 (4)	0.8323 (2)	−0.1564 (3)	0.0376 (8)	
C7	0.4973 (4)	0.8324 (2)	−0.0555 (3)	0.0352 (8)	
C8	0.5483 (4)	0.7659 (2)	0.0226 (3)	0.0465 (9)	
H8	0.5147	0.7623	0.0880	0.056*	
C9	0.6456 (4)	0.7063 (2)	0.0059 (3)	0.0510 (10)	
H9	0.6757	0.6631	0.0591	0.061*	
C10	0.6995 (4)	0.7100 (2)	−0.0902 (3)	0.0496 (10)	
H10	0.7684	0.6708	−0.1001	0.060*	
C11	0.6500 (4)	0.7715 (2)	−0.1693 (3)	0.0461 (9)	
H11	0.6852	0.7736	−0.2340	0.055*	
C12	0.1362 (4)	1.01565 (19)	0.2115 (3)	0.0352 (8)	
C13	0.2544 (3)	0.96253 (19)	0.2079 (3)	0.0326 (7)	
C14	0.3338 (4)	0.9210 (2)	0.3062 (3)	0.0437 (9)	
H14	0.4122	0.8863	0.3036	0.052*	
C15	0.2961 (4)	0.9315 (2)	0.4081 (3)	0.0518 (10)	
H15	0.3497	0.9040	0.4742	0.062*	
C16	0.1796 (5)	0.9824 (2)	0.4118 (3)	0.0529 (10)	
H16	0.1545	0.9888	0.4806	0.063*	
C17	0.0994 (4)	1.0238 (2)	0.3150 (3)	0.0470 (9)	
H17	0.0202	1.0576	0.3187	0.056*	
C18	0.0667 (4)	1.1630 (2)	−0.1946 (3)	0.0536 (10)	
H18	0.1321	1.1328	−0.2246	0.064*	
C19	−0.0109 (5)	1.2277 (3)	−0.2581 (4)	0.0704 (13)	
H19	0.0018	1.2406	−0.3298	0.085*	
C20	−0.1066 (5)	1.2730 (2)	−0.2158 (4)	0.0648 (12)	
H20	−0.1609	1.3168	−0.2582	0.078*	
C21	−0.1215 (5)	1.2530 (2)	−0.1098 (4)	0.0556 (11)	
H21	−0.1851	1.2834	−0.0780	0.067*	
C22	−0.0401 (4)	1.1868 (2)	−0.0505 (3)	0.0444 (9)	
H22	−0.0507	1.1733	0.0217	0.053*	

### Atomic displacement parameters (Å^2^)

**Table:**

	*U*^11^	*U*^22^	*U*^33^	*U*^12^	*U*^13^	*U*^23^
O2	0.0406 (14)	0.0454 (14)	0.0341 (13)	0.0068 (11)	0.0096 (11)	0.0073 (11)
Cu1	0.0346 (3)	0.0391 (2)	0.0333 (2)	0.0013 (2)	0.00662 (18)	0.00654 (19)
N1	0.0290 (15)	0.0361 (15)	0.0272 (14)	−0.0016 (13)	0.0056 (12)	0.0024 (12)
N2	0.0372 (18)	0.0337 (15)	0.0365 (17)	−0.0038 (13)	0.0021 (13)	0.0033 (13)
O1	0.0413 (15)	0.0513 (15)	0.0346 (13)	0.0052 (12)	0.0116 (11)	0.0102 (11)
C1	0.030 (2)	0.0391 (19)	0.0315 (19)	−0.0021 (16)	0.0029 (15)	0.0056 (15)
C2	0.031 (2)	0.0392 (19)	0.0285 (18)	−0.0064 (15)	0.0060 (15)	0.0012 (15)
C3	0.0327 (19)	0.0406 (19)	0.0330 (18)	−0.0091 (17)	0.0086 (15)	0.0012 (16)
C4	0.040 (2)	0.051 (2)	0.0283 (18)	−0.0080 (18)	0.0050 (15)	0.0053 (16)
C5	0.044 (2)	0.057 (2)	0.033 (2)	−0.0107 (19)	0.0149 (17)	−0.0078 (17)
C6	0.034 (2)	0.045 (2)	0.0330 (19)	−0.0064 (16)	0.0081 (16)	−0.0067 (16)
C7	0.0299 (19)	0.0406 (19)	0.0331 (19)	−0.0073 (16)	0.0051 (15)	−0.0060 (15)
C8	0.052 (2)	0.051 (2)	0.038 (2)	0.0075 (19)	0.0148 (18)	0.0039 (17)
C9	0.051 (3)	0.050 (2)	0.050 (2)	0.011 (2)	0.012 (2)	0.0022 (18)
C10	0.045 (2)	0.054 (2)	0.050 (2)	0.0042 (19)	0.0133 (19)	−0.012 (2)
C11	0.042 (2)	0.062 (2)	0.037 (2)	−0.0035 (19)	0.0154 (18)	−0.0086 (19)
C12	0.038 (2)	0.0347 (19)	0.0300 (19)	−0.0036 (15)	0.0036 (16)	0.0000 (14)
C13	0.0311 (18)	0.0366 (18)	0.0281 (17)	−0.0053 (16)	0.0046 (14)	−0.0007 (15)
C14	0.040 (2)	0.054 (2)	0.035 (2)	0.0080 (18)	0.0055 (17)	0.0047 (17)
C15	0.057 (3)	0.065 (3)	0.030 (2)	0.008 (2)	0.0063 (18)	0.0080 (18)
C16	0.067 (3)	0.063 (3)	0.031 (2)	0.008 (2)	0.0158 (19)	−0.0008 (18)
C17	0.053 (2)	0.047 (2)	0.043 (2)	0.0082 (18)	0.0175 (19)	−0.0016 (17)
C18	0.050 (3)	0.056 (2)	0.058 (3)	0.010 (2)	0.020 (2)	0.022 (2)
C19	0.070 (3)	0.075 (3)	0.070 (3)	0.020 (3)	0.026 (3)	0.041 (3)
C20	0.067 (3)	0.047 (2)	0.072 (3)	0.007 (2)	0.005 (2)	0.021 (2)
C21	0.056 (3)	0.042 (2)	0.064 (3)	0.0099 (19)	0.008 (2)	−0.004 (2)
C22	0.054 (3)	0.037 (2)	0.039 (2)	−0.0018 (18)	0.0067 (18)	−0.0044 (16)

### Geometric parameters (Å, °)

**Table:**

O2—C12	1.334 (4)	C9—C10	1.390 (5)
O2—Cu1	1.935 (2)	C9—H9	0.9300
Cu1—O1	1.899 (2)	C10—C11	1.357 (5)
Cu1—N1	1.943 (3)	C10—H10	0.9300
Cu1—N2	2.023 (3)	C11—H11	0.9300
N1—C1	1.293 (4)	C12—C17	1.393 (4)
N1—C13	1.418 (4)	C12—C13	1.408 (4)
N2—C22	1.328 (4)	C13—C14	1.387 (4)
N2—C18	1.330 (4)	C14—C15	1.381 (5)
O1—C3	1.301 (4)	C14—H14	0.9300
C1—C2	1.429 (4)	C15—C16	1.375 (5)
C1—H1	0.9300	C15—H15	0.9300
C2—C3	1.420 (4)	C16—C17	1.376 (5)
C2—C7	1.448 (4)	C16—H16	0.9300
C3—C4	1.429 (4)	C17—H17	0.9300
C4—C5	1.348 (5)	C18—C19	1.368 (5)
C4—H4	0.9300	C18—H18	0.9300
C5—C6	1.417 (5)	C19—C20	1.358 (6)
C5—H5	0.9300	C19—H19	0.9300
C6—C11	1.407 (5)	C20—C21	1.364 (5)
C6—C7	1.421 (4)	C20—H20	0.9300
C7—C8	1.410 (4)	C21—C22	1.380 (5)
C8—C9	1.370 (5)	C21—H21	0.9300
C8—H8	0.9300	C22—H22	0.9300
			
C12—O2—Cu1	111.3 (2)	C10—C9—H9	119.8
O1—Cu1—O2	176.63 (9)	C11—C10—C9	119.0 (3)
O1—Cu1—N1	92.39 (10)	C11—C10—H10	120.5
O2—Cu1—N1	84.46 (10)	C9—C10—H10	120.5
O1—Cu1—N2	91.49 (11)	C10—C11—C6	122.1 (3)
O2—Cu1—N2	91.83 (10)	C10—C11—H11	119.0
N1—Cu1—N2	168.64 (10)	C6—C11—H11	119.0
C1—N1—C13	123.2 (3)	O2—C12—C17	122.6 (3)
C1—N1—Cu1	125.7 (2)	O2—C12—C13	118.9 (3)
C13—N1—Cu1	111.1 (2)	C17—C12—C13	118.4 (3)
C22—N2—C18	117.2 (3)	C14—C13—C12	120.4 (3)
C22—N2—Cu1	121.2 (2)	C14—C13—N1	126.8 (3)
C18—N2—Cu1	121.5 (2)	C12—C13—N1	112.8 (3)
C3—O1—Cu1	127.7 (2)	C15—C14—C13	119.8 (3)
N1—C1—C2	126.4 (3)	C15—C14—H14	120.1
N1—C1—H1	116.8	C13—C14—H14	120.1
C2—C1—H1	116.8	C16—C15—C14	120.1 (3)
C3—C2—C1	121.1 (3)	C16—C15—H15	120.0
C3—C2—C7	119.4 (3)	C14—C15—H15	120.0
C1—C2—C7	119.4 (3)	C15—C16—C17	120.9 (3)
O1—C3—C2	125.2 (3)	C15—C16—H16	119.6
O1—C3—C4	116.7 (3)	C17—C16—H16	119.6
C2—C3—C4	118.1 (3)	C16—C17—C12	120.4 (3)
C5—C4—C3	122.3 (3)	C16—C17—H17	119.8
C5—C4—H4	118.9	C12—C17—H17	119.8
C3—C4—H4	118.9	N2—C18—C19	122.8 (4)
C4—C5—C6	121.7 (3)	N2—C18—H18	118.6
C4—C5—H5	119.2	C19—C18—H18	118.6
C6—C5—H5	119.2	C20—C19—C18	119.7 (4)
C11—C6—C5	121.9 (3)	C20—C19—H19	120.2
C11—C6—C7	119.6 (3)	C18—C19—H19	120.2
C5—C6—C7	118.5 (3)	C19—C20—C21	118.7 (4)
C8—C7—C6	116.3 (3)	C19—C20—H20	120.7
C8—C7—C2	123.9 (3)	C21—C20—H20	120.7
C6—C7—C2	119.8 (3)	C20—C21—C22	118.7 (4)
C9—C8—C7	122.5 (3)	C20—C21—H21	120.7
C9—C8—H8	118.8	C22—C21—H21	120.7
C7—C8—H8	118.8	N2—C22—C21	123.1 (3)
C8—C9—C10	120.5 (4)	N2—C22—H22	118.5
C8—C9—H9	119.8	C21—C22—H22	118.5

### Hydrogen-bond geometry (Å, °)

**Table:**

*D*—H···*A*	*D*—H	H···*A*	*D*···*A*	*D*—H···*A*
C18—H18···O1	0.93	2.31	2.872 (4)	119
C22—H22···O2	0.93	2.31	2.885 (4)	120
